# Global Distribution and Diversity of Marine Parmales

**DOI:** 10.1264/jsme2.ME23093

**Published:** 2024-03-23

**Authors:** Hiroki Ban, Hisashi Endo, The EukBank Team, Cédric Berney, Frédéric Mahé, Nicolas Henry, Colomban de Vargas, Akira Kuwata, Hiroyuki Ogata

**Affiliations:** 1 Bioinformatics Center, Institute for Chemical Research, Kyoto University, Gokasho, Uji, Kyoto, 611–0011, Japan; 2 Shiogama Field Station, Fisheries Resources Institute, Japan Fisheries Research and Education Agency, 3–27–5 Shinhama-cho, Shiogama, Miyagi, Japan; 3 Sorbonne Université, CNRS, Station Biologique de Roscoff, AD2M, UMR 7144, 29680 Roscoff, France; 4 CIRAD, UMR PHIM, F-34398 Montpellier, France; 5 PHIM, Univ Montpellier, CIRAD, INRAE, Institut Agro, IRD, Montpellier, France; 6 CNRS, Sorbonne Université, FR2424, ABiMS, Station Biologique de Roscoff, 29680 Roscoff, France; 7 Research Federation for the study of Global Ocean Systems Ecology and Evolution, FR2022/Tara Oceans GOSEE, 3 rue Michel-Ange, 75016 Paris, France

**Keywords:** global distribution, metabarcoding, ocean, Parmales

## Abstract

Parmales (Bolidophyceae) is a minor eukaryotic phytoplankton group, sister to diatoms, which exists as two distinct forms of unicellular organisms: silicified cells and naked flagellates. Since their discovery, many field studies on Parmales have been performed; however, their global distribution has not yet been examined in detail. We herein compiled more than 3,000 marine DNA metabarcoding datasets targeting the V4 region of the 18S rRNA gene from the EukBank database. By linking this large dataset with the latest morphological and genetic information, we provide updated estimates on the diversity and distribution of Parmales in the global ocean at a fine taxonomic resolution. Parmalean amplicon sequence variants (ASVs) were detected in nearly 90% of the samples analyzed. However, the relative abundance of parmaleans in the eukaryotic community was less than 0.2% on average, and the estimated true richness of parmalean ASVs was approximately 316 ASVs, confirming their low abundance and diversity. A phylogenetic ana­lysis divided these algae into four clades, and three known morphotypes of silicified cells were classified into three different clades. The abundance of Parmales is generally high in the poles and decreases towards the tropics, and individual clades/subclades show further distinctions in their distribution. Collectively, the present results suggest clade/subclade-specific adaptation to different ecological niches.

The order Parmales (class Bolidophyceae) comprises eukaryotic microalgae of two morphologically distinct forms: one is a naked flagellate (1–1.7‍ ‍μm in diameter) and the other has silicified cell walls (2–5‍ ‍μm in diameter) (Booth and Marchant, 1987; Guillou *et al.*, 1999; Ichinomiya *et al.*, 2011, 2016). The silicified forms were originally established as a new order, Parmales, within Chrysophyceae (Booth and Marchant, 1987). Following the first isolation of a parmalean from the Oyashio region near Japan (Ichinomiya *et al.*, 2011), phylogenetic ana­lyses revealed that parmaleans formed a monophyletic group with previously known naked flagellates (bolidophytes) (Ichinomiya *et al.*, 2011) that comprise a sister group to diatoms (Guillou *et al.*, 1999). Consequently, the order Parmales was re-established under the class Bolidophyceae (Ichinomiya *et al.*, 2016). The two forms have a phylogenetically nested relationship, and silicified strains possess the genomic potential to form flagella, suggesting that these two forms represent different stages in the life cycle of the same organisms (Ichinomiya *et al.*, 2016; Ban *et al.*, 2023). Four morphotypes of silicified parmaleans have been identified to date, with each being distinguished by the morphological features of their silicified cell walls: *Triparma*, *Tetraparma*, ‘Scaly parma’, and *Pentalamina*, the last morphotype of which has not yet been isolated (Booth and Marchant, 1987; Ban *et al.*, 2023; Sato *et al.*, unpublished).

Their life cycle and predicted phago-mixotrophic nutrient acquisition markedly contrast with those of diatoms, their closest evolutionary photo-autotrophic relatives (Ban *et al.*, 2023). Therefore, Parmales is a key eukaryotic group for understanding the physiology, ecology, and evolution of diatoms, the most successful phytoplankton group in the modern ocean (Kuwata *et al.*, 2018; Ban *et al.*, 2023). Efforts to characterize the diversity and biogeography of Parmales across space and niches are expected to provide fundamental information on differences in ecological and evolutionary strategies between diatoms and Parmales.

Based on field observations to date, the silicified form of Parmales is widely distributed from frequently-reported polar and subpolar regions, including coastal sites (Booth *et al.*, 1980, 1981; Silver *et al.*, 1980; Nishida, 1986; Taniguchi *et al.*, 1995; Komuro *et al.*, 2005; Konno *et al.*, 2007; Ichinomiya *et al.*, 2010, 2019; Ichinomiya and Kuwata, 2015; Luan *et al.*, 2018; Hoshina *et al.*, 2021a, 2021b, 2021c), to the tropics (Kosman *et al.*, 1993; Bravo-Sierra and Hernández-Becerril, 2003; Fujita and Jordan, 2017). However, these observations were based on microscopic ana­lyses of the silicified cell wall morphology and, thus, naked flagellates were missed. There were also potential issues regarding cryptic species (Bickford *et al.*, 2007) and variations within a single species due to morphological phenotypic plasticity (Konno *et al.*, 2007). Therefore, an accurate and consistent taxon identification method is needed to quantify the abundance and diversity of Parmales.

DNA metabarcoding targeting the V4 or V9 region of the 18S rRNA gene has recently become an effective method to examine the eukaryotic diversity and community composition of the ocean (De Vargas *et al.*, 2015; Massana *et al.*, 2015; Cordier *et al.*, 2022). DNA metabarcoding bypasses microscopic taxonomy identification and provides abundant comprehensive data that may resolve the issues associated with microscopic observations. Previous studies investigated the distribution of Parmales using the V9 metabarcoding dataset produced by the *Tara* Oceans expedition (Ichinomiya *et al.*, 2016) and the V4 metabarcoding datasets from multiple studies covering coastal, Arctic, and Antarctic oceans not represented in *Tara* Oceans data (Kuwata *et al.*, 2018). These studies characterize the global distribution of Parmales in the ocean, revealing their consistently low frequency in microeukaryotic communities and suggesting that each clade of Parmales has its own distribution pattern. However, the data used by Ichinomiya *et al.* (2016) from the *Tara* Oceans expedition do not cover coastal areas and some oceanic regions, such as the Indian Ocean and Antarctic Sea, while data used by Kuwata *et al.* (2018) underrepresent the Southern Hemisphere. Additionally, when these studies were conducted, sequence information on isolated strains was only available for one clade, *Triparma*, of the four morphotypes.

In the present study, we used the EukBank database, which provides the largest dataset for DNA metabarcodes targeting the 18S rRNA gene V4 region (Kaneko *et al.*, 2023). The EukBank project consolidates multiple datasets originating from various sampling projects, including *Tara* Oceans (Pesant *et al.*, 2015; Sunagawa *et al.*, 2020), Malaspina (Logares *et al.*, 2020), and the Australian Microbiome (Brown *et al.*, 2018). This dataset comprises more than 15,000 DNA metabarcoding samples targeting the 18S rRNA gene V4 region, with samples derived from various biomes, such as marine, freshwater, and soil biomes. After appropriate filtering, we compiled more than 3,000 marine samples from pole-to-pole oceanic regions, including coastal areas. We associated these data with current knowledge on Parmales, including information on recent isolates from *Triparma*, *Tetraparma*, and ‘Scaly parma’ (Yamada *et al.*, 2020; Ban *et al.*, 2023). The present results quantitatively describe the diversity and distribution of Parmales at a fine taxonomic resolution.

## Materials and Methods

### EukBank data preprocessing

The EukBank database provides amplicon sequencing variant (ASV) sequences and their taxonomic annotations, and also includes table data representing the read counts of ASVs in each sample with metadata. Detailed information on the preprocessing of raw data is provided in Kaneko *et al.* (2023).

We initially selected seawater samples with metadata and a total read count higher than 10,000. We then filtered samples based on the availability of sampling site information on latitude, longitude, and depth. During this process, samples without specific depth information were also retained if the range of sampling depth was obtained from other information (Supplementary Information). Depth information was finally classified as either the surface layer (0–10 m) or euphotic zone (10–200 m). We subsequently retained only samples (depth 0–200 m) with a lower limit of the size fraction of less than 1‍ ‍μm. This size threshold was selected with the expectation that it would allow all parmalean morphological forms to be captured on the filter. We then categorized samples based on their seafloor depth at the sampling sites; sites shallower than 200‍ ‍m were defined as coastal ocean sites, while deeper sites were defined as open ocean sites. The depths of the sampling sites were calculated by interpolating depth data from the global relief model (ETOP 1) (Amante, 2009) using latitude and longitude information with the Julia package “Interpolations.jl”. We finally collected 3,200 marine samples, 1,633 of which were coastal ocean samples and 1,567 were open ocean samples (Fig. S1).

We also obtained 432 ASV sequences that were annotated as “Bolidophyceae” from all EukBank ASV sequences.

### Phylogenetic ana­lyses and ASV annotation

**Fig. 1.**
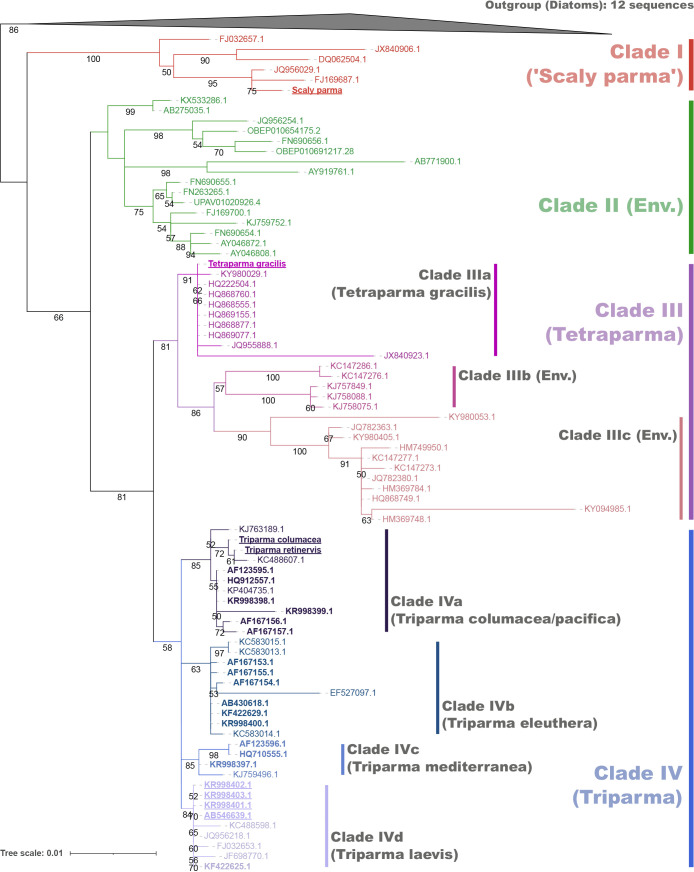
Phylogenetic tree of 18S rRNA genes. Maximum likelihood phylogenetic tree of the full-length 18S rRNA genes of Parmales and diatoms (outgroup). IDs of isolates are in bold and, if isolates are of the silicified form, IDs are underlined. Bootstrap values ≥50 are noted on the nodes. Clades/subclades were separated based on the tree topology.

We collected full-length parmalean 18S rRNA gene sequences from the SILVA database (categorized as “Bolidomonas”; accessed Jun 2023) (Quast *et al.*, 2012) and from published work (Ban *et al.*, 2023). We added some diatom sequences as outgroups to the dataset and then removed previously reported chimeric sequences (Ichinomiya *et al.*, 2016) and sequences shorter than 900 bp. We aligned and masked the sequences using the “ssu-align” and “ssu-mask” commands of SSU-ALIGN (v.0.1.1) (Nawrocki, 2009) with default parameters. A maximum likelihood tree was estimated with a GTR+G+F model and 1,000 bootstrap replicates using RAxML-ng (v.1.0.2) (Kozlov *et al.*, 2019). We defined clades and subclades based on the topology of the estimated phylogenetic tree according to previous studies (Ichinomiya *et al.*, 2016; Kuwata *et al.*, 2018) (Fig. 1).

ASVs were assigned their phylogenetic placement on the estimated reference tree. Full-length 18S rRNA gene sequences were aligned to make a multiple sequence alignment using the “ssu-align” command with default parameters; however, since default masking causes a loss in resolution for distinguishing ASVs, masking of the alignment was performed using the “ssu-mask” command with the “--rfonly” option. The sequences of 432 parmalean ASVs were also aligned and masked using the “ssu-align” command with default parameters and the “ssu-mask” command with the previously produced mask file from full-length 18S rRNA gene alignments. Model parameters for phylogenetic placements were evaluated with the full-length 18S rRNA gene alignment and the estimated tree using RAxML-ng (Kozlov *et al.*, 2019) with the “--evaluate” option (v.1.0.2) specifying the GTR+G+F model. Phylogenetic placement was performed using EPA-ng (Barbera *et al.*, 2019) under the evaluated model with the ASV alignment as the query and the maximum likelihood tree and full-length 18S rRNA gene alignment as the reference.

To annotate each ASV into a clade/subclade, we used the “extract” command of gappa (v.0.8.4) (Czech *et al.*, 2020). ASVs were annotated into Clades I–IV, “basal_branches”, and “outgroup”. We herein prioritized EukBank’s taxonomy; therefore, ASVs annotated as “outgroup” were treated as a Parmales origin, while those annotated as “basal_branches” and “outgroup” were grouped together as “uncertain” sequences of Parmales. At the subclade level, Clade III was divided into Clades IIIa–IIIc, and Clade IV into Clades IVa–IVd (Fig. 1). ASVs annotated as Clade III or IV at the clade level, but as “basal_branches” at the subclade level were re-annotated as “Clade III uncertain” or “Clade IV uncertain”, respectively.

Parmalean ASV sequences were also aligned to full-length 18S rRNA gene sequences using vsearch (v.2.22.1) (Rognes *et al.*, 2016) for sequence similarity searches.

### Ecological ana­lyses

Rarefaction curves were obtained by plotting the expected count of ASVs calculated under the assumption that all reads from 3,200 samples were pooled and then sub-sampled. The number of reads sub-sampled increased in increments of 10,000. The slopes of the rarefaction curves were calculated from the last data points and their predecessors.

Preston’s log-normal distribution (Preston, 1948) was used to estimate the completeness of sampling by fitting a left-truncated normal distribution to the log2-transformed total counts of each ASV in 3,200 samples using function “prestondistr” in the R package “vegan” via Julia package “RCall.jl”.

The relationships between latitude and parmalean ASV abundances were visualized by Locally Estimated Scatterplot Smoothing (LOESS; span=0.8) using the Julia package “Loess.jl”. Confidence intervals were computed using 100 bootstrap resampling iterations.

To characterize the habitats of each clade and subclade, the weighted average temperature (WAT) was used as the index for clade/subclade temperature preferences using the relative abundance in 2,002 of the 3,200 samples for which temperature information was available. This index was calculated using the following equation:

$$WAT_{k}=\frac{\sum w_{ki}t_{i}}{\sum w_{ki}}$$

where *WAT_k_* is the weighted average temperature index of clade/subclade *k*, and *w_ki_* and *t_i_* are the relative abundances of clade/subclade *k* and water temperature in sample *i*, respectively.

Plots were generated using the Julia packages “Makie.jl” (Danisch and Krumbiegel, 2021) and “GeoMakie.jl”.

### Statistical ana­lysis

Regarding each clade and subclade, the Mann-Whitney U test (Mann and Whitney, 1947) was employed using the Julia package “HypothesisTests.jl” to evaluate whether a difference existed in relative abundance between coastal ocean samples (*n*=1,633) and open ocean samples (*n*=1,567). The results obtained were further validated using the rank-biserial correlation (RBC), which is the effect size of the Mann-Whitney U test (Wendt, 1972; Kerby, 2014). Positive RBC values indicated a preference for the coastal ocean and negative values for the open ocean; the absolute value (0–1) indicated the strength of the preference.

## Results

### Phylogenetic ana­lyses

The maximum likelihood tree (Fig. 1) based on the phylogenetic ana­lysis of full-length 18S rRNA gene sequences showed the clear grouping of parmalean sequences into four clades (Clade I–IV), which is consistent with previous findings (Kuwata *et al.*, 2018). Clade I is the most basal clade, containing ‘Scaly parma’, which are morphologically distinct from other parmalean taxa (Ban *et al.*, 2023; Sato *et al.*, unpublished). Clade II consists only of environmental sequences and does not include any sequences from isolates of either silicified forms or naked flagellates. Clade III is divided into three subclades based on topology (Clade IIIa–IIIc). Clade IIIa contains a silicified isolate sequence (*Tetraparma gracilis*) and a sequence of unknown morphology (KY980029: *Triparma pacifica* isolate NY13S_157), while Clade IIIc contains two sequences of unknown morphology (KY980053: *T. pacifica* isolate NY13S_197; KY98405: *T. pacifica* isolate BH65_151). Clade IIIb consists only of environmental sequences. The taxonomic annotation of *Triparma* for the three sequences of unknown morphology in Clade III may be the misannotation of samples that properly belong to *Tetraparma*. Clade IV is also divided into four subclades (Clade IVa–IVd). Clade IVa and Clade IVd contain sequences from silicified isolates and naked flagellate isolates, with *Triparma columacea* and *Triparma retinervis* representing the silicified form and *T. pacifica* RCC205 (HQ912557) representing the naked flagellate for Clade IVa. In Clade IVd, *Triparma laevis* f. *inornata* (AB546639) represents the silicified form, while *Triparma sp.* RCC1657 (KF422625) represents the naked flagellate. Clades IVb and IVc do not have any sequences from silicified form isolates, with *Triparma eleuthera* (KR998400) and *Triparma mediterranea* (KR998397) representing naked flagellates for Clades IVb and IVc, respectively.

As a result of the phylogenetic placement of ASVs in the reference maximum likelihood tree, 86.3% of ASVs were annotated into one of the four clades (Table 1). There were no ASVs that showed the best match with outgroup sequences in the sequence similarity search, suggesting that all ASVs were derived from Parmales. ASVs that were annotated into clades, as well as those that were not, had sequence identities of 91.7 and 83.1%, respectively, on average with their closest matching full-length 18S rRNA gene sequences (Fig. S2). Clade II, the environmental clade, contained the most annotated ASVs, followed by Clades I, III, and IV (Table 1).

### Global marine dataset of parmalean ASVs

**Fig. 2.**
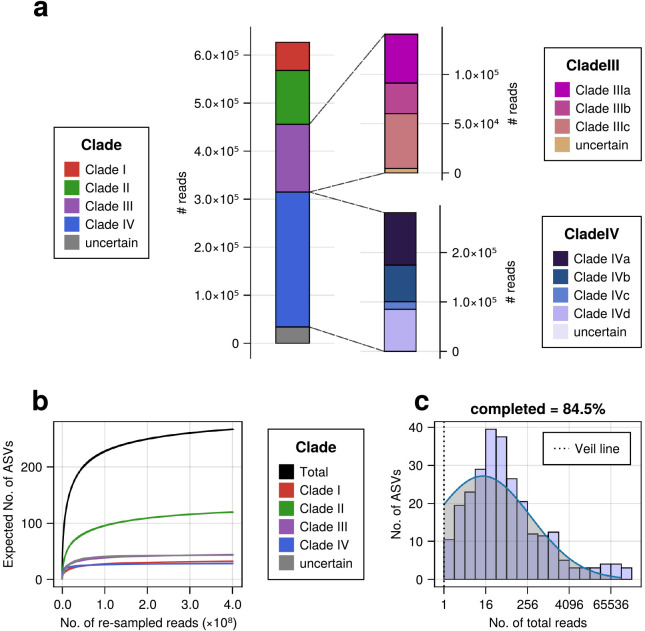
Overview of the parmalean ASV dataset. (a) The number of reads of parmalean ASVs in each clade from 3,200 samples. (Top right) The number of reads of parmalean ASVs in Clade III. (Bottom right) The number of reads of parmalean ASVs in Clade IV. (b) Rarefaction curve, representing parmalean ASV richness. Each curve shows the expected number of parmalean ASVs against the number of re-sampled reads for each clade. The slopes of each curve are listed in Table S1. (c) Preston’s log-normal distribution of parmalean ASVs. The x-axis is transformed to log2. The histogram represents the frequency of actual parmalean ASVs binned by abundance in octaves. The blue line indicates the fitted left-truncated normal distribution. The left side of Preston’s Veil line (dashed black vertical line) corresponds to ASVs that did not appear in the samples. Total parmalean ASV richness was extrapolated to ASVs on this side.

After filtering samples from the EukBank database, we obtained 3,200 marine water samples containing approximately 402 million reads. Although the coastal ocean samples were predominantly from Europe and Australia, the open ocean samples widely ranged from pole to pole, with some exceptions (*e.g.*, the West Pacific) (Fig. S1). These samples covered oceanic regions that had not appeared in previous studies (Ichinomiya *et al.*, 2016; Kuwata *et al.*, 2018) and alleviated the data bias issue. Among 432 EukBank ASVs, 267 ASVs appeared at least once and accounted for approximately 630,000 of the total reads. Overall, 94.6% of parmalean ASV reads were assigned to four clades (Fig. 2a), of which Clade IV was the most dominant, followed by Clades III, II, and I (Fig. 2a). This order of abundance was not consistent with the order of diversity inside the clades (Table 1). Within Clade III, Clade IIIc was slightly more abundant than Clade IIIa, with Clade IIIb being the least abundant (Fig. 2a). Within Clade IV, Clade IVa was the most abundant, followed by Clades IVd and IVb, with Clade IVc being markedly less abundant (Fig. 2a). A rarefaction ana­lysis indicated that the ASV richness of all global ocean parmaleans was far from saturation (Fig. 2b). Clade II in particular had the largest slope (Table S1). The fitted Preston model (blue line in Fig. 2c) extrapolated true parmalean ASV richness to 315.85 ASVs, indicating that the analyzed samples uncovered ~84.5% of parmalean ASVs in the global ocean (right side of the veil line in Fig. 2c).

### Oceanic distribution of parmalean ASVs

**Fig. 3.**
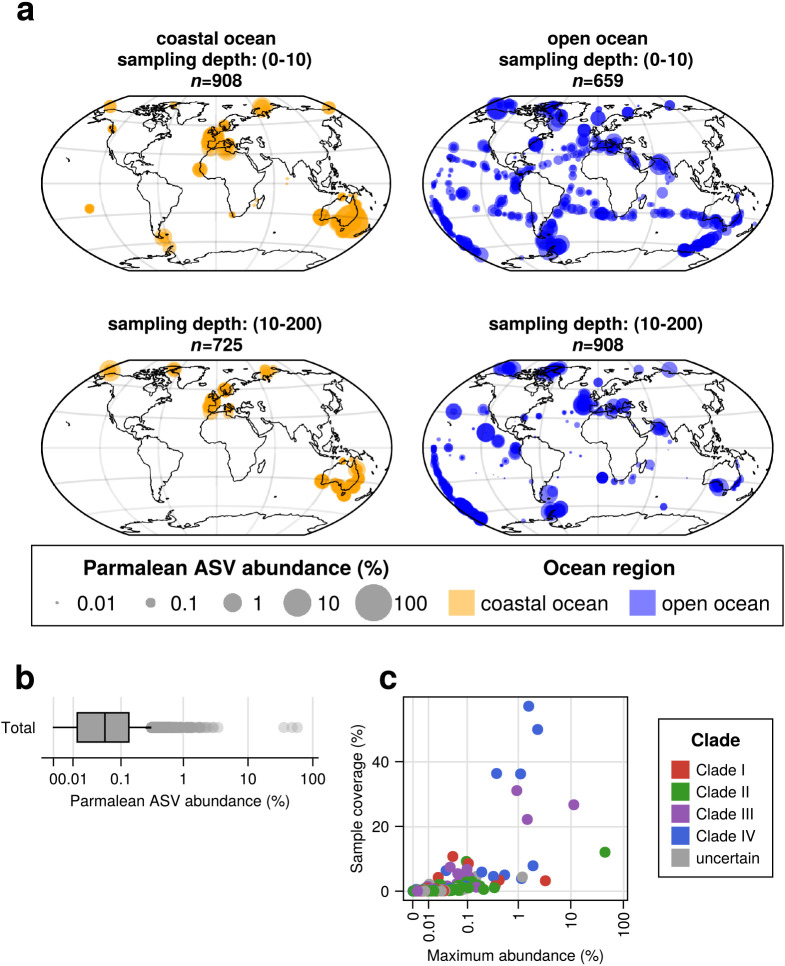
Overview of the parmalean ASV distribution in the global ocean. (a) Global distribution of parmalean ASVs. (Top left) Coastal ocean surface layer, (bottom left) Coastal ocean euphotic zone, (top right) Open ocean surface layer, and (bottom right) Open ocean euphotic zone. Dot sizes are scaled to log10 (“parmalean ASV abundance”×10,000+1) at each sample. (b) Parmalean ASV abundance of each sample. The x-axis is scaled with the pseudolog10 function. (c) Sample coverage and the maximum relative abundance of each ASV. The x-axis is scaled with the pseudolog10 function.

Parmalean ASVs were widely distributed across the global ocean in both coastal and open oceans from the poles to the tropics (Fig. 3a). Parmalean ASVs appeared in 89.1% of the samples, indicating their wide distribution. However, the relative abundance of parmalean ASVs in the microeukaryotic community was generally low, with a median of 0.05% and an average of 0.16% (Fig. 3b). Nevertheless, there were samples where parmalean ASVs accounted for a markedly large percentage of the community (three outliers in Fig. 3b). In three samples taken on the same day and location in Botany Bay of Sydney, Australia, parmalean ASVs contributed to 58.2, 48.1, and 36.5% of the microeukaryotic community (SRA Run: SRR8820967, SRR8820804, and SRR8820815, respectively, from the Australian Microbiome). ASVs with a high sample coverage (*i.e.*, the percentage of samples in which they were detected had a slightly high maximum relative abundance) (Fig. 3c). However, an ASV that was dominant in one sample was not necessarily widely distributed.

**Fig. 4.**
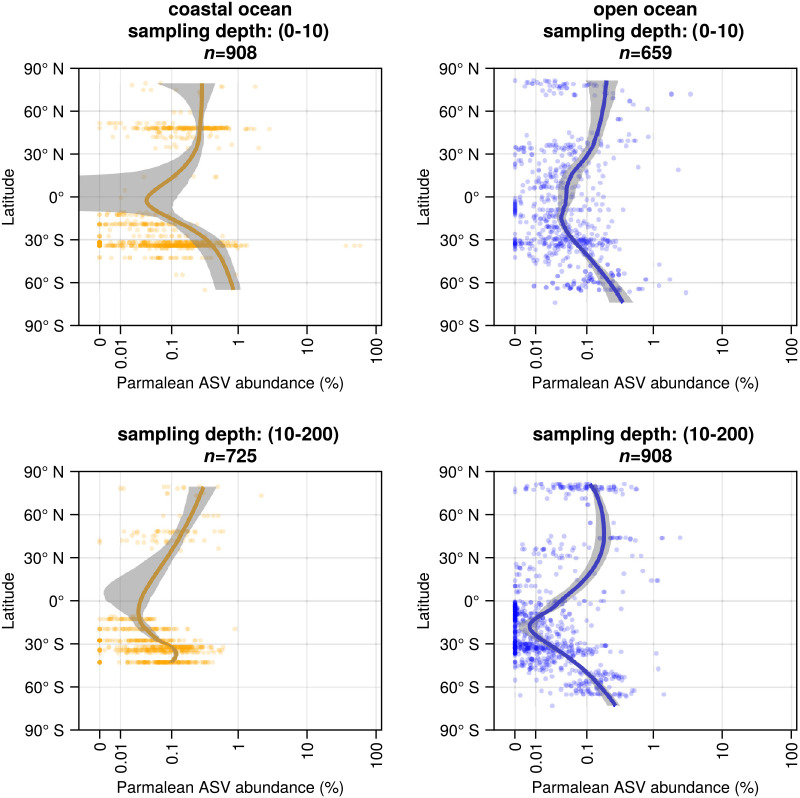
Latitudinal trends of parmales ASV abundance (Top left) Coastal ocean surface layer, (Bottom left) Coastal ocean euphotic zone, (Top right) Open ocean surface layer, and (Bottom right) Open ocean euphotic zone. Shaded areas represent 90% confidence intervals.

The relative abundance of parmalean ASVs across latitudes showed a clear previously undescribed pattern that decreased from the poles to tropics, while they were detected in the coast and open oceans across the surface and euphotic zones (Fig. 4). In the open ocean, the relative abundance of parmalean ASVs decreased more markedly in the tropics in the euphotic layer than in the surface layer (Fig. 4).

### Global diversity pattern of parmalean ASVs.

**Fig. 5.**
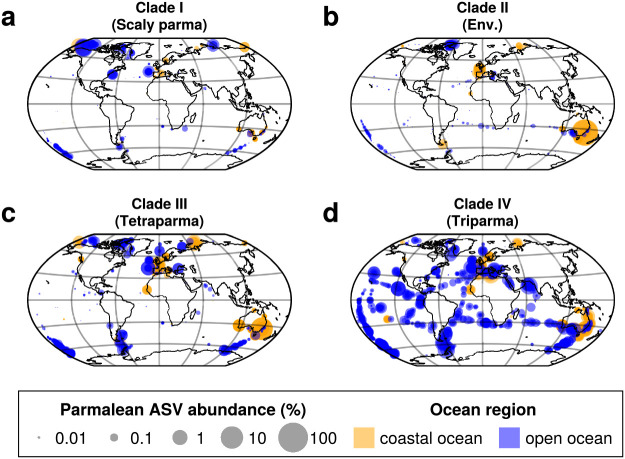
Global distribution of parmalean ASVs in each clade. Samples from all depths are shown in single plots. Orange dots indicate coastal ocean samples and blue dots indicate open ocean samples. Dot sizes are scaled to log10 (“parmalean ASV abundance”×10,000+1) at each sample. (a) Clade I (‘Scaly Parma’), (b) Clade II (environmental clade), (c) Clade III (*Tetraparma*), and (d) Clade IV (*Triparma*).

**Fig. 6.**
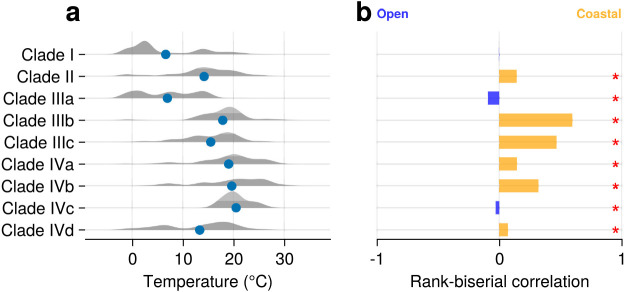
Characteristics of the distribution of clades/subclades. (a) Temperature preference of each clade/subclade. Blue dots represent the respective WAT indices. Shaded areas are violin plots of temperature weighted by frequency in each sample. (b) Preference between the coastal ocean and open ocean of each clade/subclade. The bar plot indicates the rank-biserial correlation, which is an effect size of the Mann-Whitney U test. Positive RBC values indicate a preference for the coastal ocean, negative values indicate a preference for the open ocean, and the absolute value (0–1) indicates the strength of the preference. Asterisks indicate the significance of differences in the test for each clade/subclade (*P*<0.05). Detailed results of the Mann-Whitney U test are shown in Table S2.

Although all Parmales clades were distributed in both the coastal and open oceans, distinct distribution patterns emerged for the clades (Fig. 5). Clade I (‘Scaly parma’) appeared to be biased towards subarctic to polar regions (Fig. 5a). The WAT index of Clade I was 6.64°C, suggesting a preference for lower temperatures (Fig. 6a and S3a). Clade II (environmental clade) was rare in the tropics, but was widely distributed through the mid- and high-latitude areas (Fig. 5b). The WAT index of Clade II was 14.2°C, which was higher than that of Clade I (Fig. 6a and S3b).

**Fig. 7.**
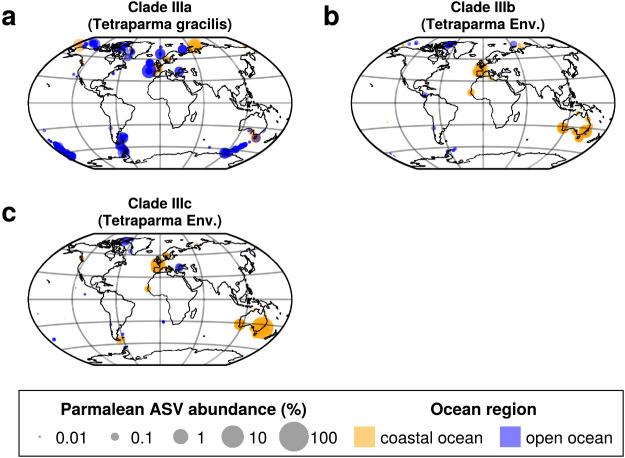
Global distribution of parmalean ASVs in each subclade of Clade III. Same legend as in Fig. 5. (a) Clade IIIa (*Tetraparma gracilis*), (b) Clade IIIb (environmental clade), and (c) Clade IIIc (*Tetraparma*).

We conducted a detailed subclade ana­lysis of Clade III (*Tetraparma*) and Clade IV (*Triparma*) (Fig. 5c and d). The three subclades of Clade III showed clearly distinct distribution patterns between Clade IIIa and Clade IIIb/Clade IIIc. Clade IIIa was distributed in both the coastal and open oceans from the subarctic to polar regions, suggesting a preference for cold water (Fig. 6a and 7a). Clades IIIb and IIIc exhibited a strong bias towards coastal oceans (Fig. 7b and c), with the RBC values of Clades IIIb and IIIc being 0.598 and 0.468, respectively (Fig. 6b, S5d, S5e, and Table S2).

**Fig. 8.**
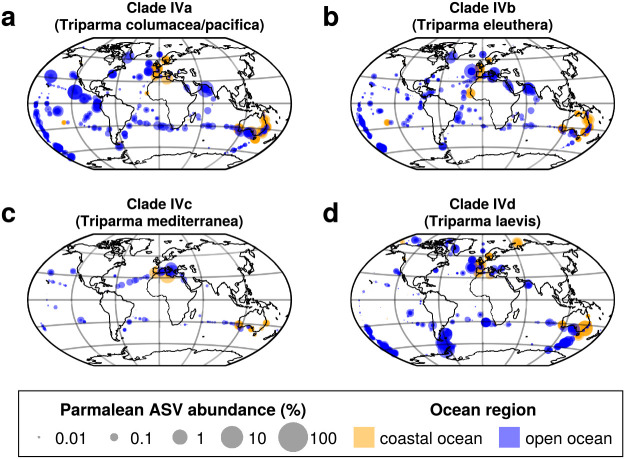
Global distribution of parmalean ASVs in each subclade of Clade IV. Same legend as in Fig. 5. (a) Clade IVa (*Triparma columacea* and *Triparma pacifica*), (b) Clade IVb (*Triparma eleuthera*), (c) Clade IVc (*Triparma mediterranea*), and (d) Clade IVd (*Triparma laevis*).

Clades IVa, IVb, and IVd were very widely distributed, with each being detected in more than 50% of samples (Fig. 8a, b, and d). In contrast, Clade IVc was narrowly distributed and present in only 8.25% of samples. Clade IVc was only found in the mid-latitude zones of both hemispheres, such as the Mediterranean Sea (Fig. 8c), and in a narrower range of water temperatures (Fig. 6a and S4f). Clades IVa and IVb exhibited similar distribution patterns, being relatively rare in polar regions, but still widely distributed (Fig. 8a and b); the WAT indices of the two clades were 19.0 and 19.7°C, respectively (Fig. 6a). The RBC values of Clades IVa and IVb were 0.146 and 0.320, respectively, with Clade IVb showing a stronger preference for the coastal oceans than Clade IVa (Fig. 6b, S5f, S5g, and Table S2). Clade IVd was widely distributed, but less abundant in the tropics, in contrast to Clade IVa and Clade IVb (Fig. 8d). The WAT index of Clade IVb was 13.3°C, suggesting a preference for cold oceans (Fig. 6a and S4g).

## Discussion

Using larger DNA metabarcoding datasets than those previously employed (Ichinomiya *et al.*, 2016; Kuwata *et al.*, 2018), we found that Parmales was broadly distributed from coastal to open oceans and from pole to pole (Fig. 3a). However, its relative abundance in the microeukaryotic community was less than 0.2% on average, as previously reported (Ichinomiya *et al.*, 2016; Kuwata *et al.*, 2018). Therefore, Parmales may be regarded as a cosmopolitan phytoplankton group belonging to a rare biosphere (Lynch and Neufeld, 2015). Nevertheless, its relative abundance sometimes exceeds 1% (up to approximately 60%) (Fig. 3b), and it may occasionally play important ecological roles.

The predicted true richness of Parmales in the global ocean was approximately 316 ASVs. This low level of diversity is in marked contrast to that of their sister group, diatoms, which are estimated to have approximately 100,000 ribotypes of the V9 region in the global ocean, methodological differences notwithstanding (Malviya *et al.*, 2016). Therefore, Parmales is a minor group of eukaryotes with respect to diversity. The fitted Preston model suggests that the ASVs collected in the present study covered 84.5% of the entire diversity of Parmales (Fig. 2c).

Previous field observations on silicified Parmales cells showed a strong relationship between water temperature and their distribution (Ichinomiya and Kuwata, 2015; Ichinomiya *et al.*, 2019; Hoshina *et al.*, 2021c). We revealed that the relative abundance of Parmales increased with latitude (Fig. 4), which supports a relationship between water temperature and the distribution of Parmales; however, our ana­lyses did not distinguish silicified forms from naked flagellates and, thus, differed from previous studies.

The phylogenetic ana­lysis of full-length 18S rRNA gene sequences recovered four clades of Parmales, as previously reported (Kuwata *et al.*, 2018; Ban *et al.*, 2023) (Fig. 1). These sequences also include recent isolates of silicified forms (Ban *et al.*, 2023), particularly ‘Scaly parma’ and *T. gracilis*. Together with a previous ana­lysis (Ban *et al.*, 2023), our study confirmed the phylogenetic positions of ‘Scaly parma’ in Clade I and *Tetraparma* in Clade III (Fig. 1). Isolates of *Triparma* belonged to Clade IV. Clade II consisted only of environmental sequences with moderate levels of relative abundance (Fig. 2a) and a relatively high level of diversity (Fig. 2b and Table 1). *Pentalamina* (Booth and Marchant, 1987) is a genus of Parmales with a distinct morphology; however, there have been no isolates or 18S rRNA sequences from this genus. Since each silicified form of parmaleans in Clades I, III, and IV has a distinct morphology and *Pentalamina* also has a distinct morphology from other parmaleans, *Pentalamina* is the prime candidate for a member of environmental Clade II. However, unidentified parmalean species may also be members of Clade II or, in consideration of the genetic diversity of Clade II (Fig. 2b), *Pentalamina* and unidentified species may both be members of Clade II. *Pileolosphaera longistirpes* may be such a member because it was initially identified as a parmalean based on its morphology (Tanimoto *et al.*, 2003). However, a subsequent study showed that its plates were made of calcium rather than silica (Meier *et al.*, 2014), leaving it as‍ ‍a‍ ‍remote candidate for examination by isolation and sequencing.

Individual clades/subclades showed distinct distribution patterns (Fig. 5, 6, 7, 8, S3, S4, S5, and Table S2), suggesting their adaptation to different ecological niches. Clade I (‘Scaly parma’) showed a strong preference for high-latitude regions and lower temperatures (Fig. 5a and 6a), which is consistent with previous findings (Kuwata *et al.*, 2018) and our observation of the lone ‘Scaly Parma’ isolate derived from the cold waters of the Sea of Okhotsk (Ban *et al.*, 2023). Clade I was the least abundant among the four clades (Fig. 2a). The lack of field observations (apart from the single isolate) may be explained by this low abundance.

Clade II (environmental clade) also showed a preference for cold water, though not to the extent of Clade I (Fig. 5b and 6a). In samples in which Parmales dominated in Botany Bay, Sydney, Australia, the most frequent ASV belonged to Clade II. Therefore, Clade II members may have the ability to cause blooms. As described above, we speculate that *Pentalamina* belongs to Clade II. The silicified form of *Pentalamina* has only been reported from the Antarctic Ocean (Kuwata *et al.*, 2018), which overlaps with the distribution of Clade II.

Clade III (*Tetraparma*) and Clade IV (*Triparma*) showed distinct distribution patterns when divided into subclades (Fig. 6, 7, and 8). Clade IIIa (*T. gracilis*) preferred cold water (Fig. 6a and 7a), which is consistent with previous findings showing that the silicified form of *T. gracilis* was frequently observed in cold water (Ichinomiya *et al.*, 2019; Hoshina *et al.*, 2021c), while Clades IIIb and IIIc showed different distribution patterns; they were preferentially distributed in coastal areas (Fig. 6b, 7b, 7c, S5f, S5g, and Table S2).

Clade IVc (*T. mediterranea*) preferred the mid-latitude of both hemispheres (Fig. 8c). Previous studies proposed that the distribution of *T. mediterranea* was mostly restricted to the Mediterranean Sea (Ichinomiya *et al.*, 2016; Kuwata *et al.*, 2018). The present study revealed that the mid-latitude distribution is a feature of Clade IVc, suggesting that parmaleans of this clade are restricted to a narrow temperature range (Fig. 6a). Clades IVa, IVb, and IVd were widely distributed and four of the highest sample coverage ASVs were from these clades (Fig. 3c). Clade IVd (*T. laevis*) showed a preference for cold water, as previously reported (Fig. 6a and 8d) (Ichinomiya *et al.*, 2016; Kuwata *et al.*, 2018), while Clade IVa (*T. columacea*, *T. pacifica*) and Clade IVb (*T. eleuthera*) showed a preference for warmer water (Fig. 6a, 8a, and 8b). This result is to some extent consistent with previous growth experiments; silicified form isolates of *T. laevis* f. *inornata*, *T.* laevis f. *longispina*, and *Triparma strigata* (Clade IVd) grew in cold water, but not in water warmer than 15°C (Ichinomiya and Kuwata, 2015), while the naked flagellate isolate of *T. eleuthera* (Clade IVb) grew at 16–24°C (Stawiarski *et al.*, 2016). Previous field observations from the subarctic Pacific also showed that the silicified forms of *T. laevis* f. *inornata*, *T. laevis* f. *longispina*, *T. strigata*, and *Triparma verrucosa* (Clade IVd) preferred cold water, which was generally restricted to less than 8°C, while the silicified forms of *T. columacea* and *T. retinervis* (Clade IVa) preferred warm water, which had a wider range of temperatures in the warm direction of up to approximately 12°C (Hoshina *et al.*, 2021c). However, the ASVs of Clades IVa and IVd also appeared in warmer waters (Fig. 6a and S4). This inconsistency may be explained by diversity within subclades, such as the existence of populations that prefer warmer water, or the possibility that they shift to a naked flagellate form in warmer water and evade microscopic observations. Field observations on slightly different forms of *T. laevis*, *T. columacea*, and *T. retinervis* in subtropics to tropics support the former idea (Kosman *et al.*, 1993; Bravo-Sierra and Hernández-Becerril, 2003; Fujita and Jordan, 2017).

With the exception of Clades IIIb and IIIc (coastal groups), all clades/subclades commonly appeared in both coastal and open oceans (Fig. 5, 6b, 7, 8, S5, and Table S2), suggesting that many parmaleans have the ability to adapt to both eutrophic coastal oceans and nutrient-depleted open ocean. Parmales may switch between silicified photoautotrophic and naked flagellated phago-mixotrophic stages in their life cycle (Ichinomiya *et al.*, 2016; Ban *et al.*, 2023), and mixotrophs are generally considered to widen their niche by alternating their trophic strategies (Endo *et al.*, 2018; Xu *et al.*, 2022). Therefore, the present results corroborate the idea that the cosmopolitan distribution of parmaleans may be explained by their life cycle strategy (Ban *et al.*, 2023).

By integrating the large dataset produced by EukBank with previous morphological and genetic information, we firmly established that Parmales is a cosmopolitan, but rare group of microeukaryotes that occasionally make blooms. The mapping of morphological features onto the phylogenetic tree revealed still sparse, but consistent signals supporting the correspondence between the clades and different morphologies. Different clades display distinct spatial distributions, suggesting ecological niche differentiations during the evolutionary process of Parmales. We consider the biogeography of different clades of Parmales revealed in this study to provide insights into the physiology, ecology, and evolution of Parmales.

### Data availability

Data from the EukBank project are available from a previous study (Kaneko *et al.*, 2023). Data supporting the present results are available at GenomeNet FTP: https://www.genome.jp/ftp/db/community/parmales_diatoms.

## Citation

Ban, H., Endo, H., The EukBank Team, Kuwata, A., and Ogata, H. (2024) Global Distribution and Diversity of Marine Parmales. *Microbes Environ ***39**: ME23093.

https://doi.org/10.1264/jsme2.ME23093

## Supplementary Material

Supplementary Material

## Figures and Tables

**Table 1.** Counts of parmalean ASVs in each annotation category.

Annotation	No. of parmalean ASVs in all EukBank samples	No. of parmalean ASVs in 3,200 marine samples
Clade I	76	32
Clade II	214	120
Clade III	50	44
Clade IV	33	28
Uncertain	59	43
**Total**	**432**	**267**
