# Supplementary material for: Global Distribution and Diversity of Marine Parmales
Source: Microbes Environ. 2024 Mar 23;39(1):ME23093. doi: 10.1264/jsme2.ME23093 (PMC10982110; doi:10.1264/jsme2.ME23093)
Supplement: Supplementary file 1 — Supplementary Material [file 39_23093_s1.pdf]

Supplementary information: Global distribution and diversity of marine Parmales

Hiroki Ban[1], Hisashi Endo[1], The EukBank Team [†], Akira Kuwata[2], Hiroyuki Ogata[1,*]

Affiliations:

1. Bioinformatics Center, Institute for Chemical Research, Kyoto University, Gokasho, Uji, Kyoto, 611-0011, Japan
2. Shiogama Field Station, Fisheries Resources Institute, Japan Fisheries Research and Education Agency, 3-27-5 Shinhama-cho, Shiogama, Miyagi, Japan

***Corresponding author**:

H. Ogata, E-mail: ogata@kuicr.kyoto-u.ac.jp, Phone: +81-774-38-3270

† Co-authors from The EukBank Team are listed in The EukBank Team Members and Affiliations Section.

This PDF file includes:

Supplementary Note

Table S1, S2

Fig. S1 to S5

Supplementary Note

Depth data preprocessing

In this study, in order to maximize the number of samples for analysis, depth categories in metadata and information from the original papers were used for samples without sampling depth values. The information was used to classify the samples into two categories: 0 m to less than 10 m (surface layer) and 10 m to less than 200 m (euphotic zone). Specifically, the categorization proceeded as follows. Initially, all samples with depth values were systematically categorized to their respective depths. Then we eliminated samples that lacked both depth and depth category data. For the samples without depth data but with the depth category data being '[SRF] surface water layer (ENVO_00010504)', only samples under project pohem (Ramond et al., 2019, 2021) were retained and categorized to the surface layer, because the original papers documented that these samples were collected at 0–5 m. Other samples were removed because they either deviated from the specified range, lacked the necessary descriptions in the original papers, or lacked original papers. For the samples without depth data but with the depth category data being '[DCM] deep chlorophyll maximum layer (ENVO_01000326)', all samples are retained and categorized to the euphotic zone. For the samples without depth data but in other depth categories, all samples were removed.

Supplementary References

Ramond, P., Siano, R., Schmitt, S., De Vargas, C., Marié, L., Memery, L., et al. (2021). Phytoplankton taxonomic and functional diversity patterns across a coastal tidal front. *Sci. Rep.* 11, 2682. doi: 10.1038/s41598-021-82071-0.

Ramond, P., Sourisseau, M., Simon, N., Romac, S., Schmitt, S., Rigaut-Jalabert, F., et al. (2019). Coupling between taxonomic and functional diversity in protistan coastal communities. *Environ. Microbiol.* 21, 730–749. doi: 10.1111/1462-2920.14537.

Table S1. Slopes of each rarefaction curve.

**Annotation**	**Slope**
Total	5.22e-8
Clade I	7.45e-9
Clade II	2.98e-8
Clade III	9.94e-9
Clade IV	2.48e-9
Uncertain	2.48e-9

Table S2. Results of Mann-Whitney U test comparing relative abundance between coastal ocean and open ocean.

Annotation	*p*-value	Rank-biserial correlation
Clade I	0.9614	-0.002
Clade II	<0.00000	0.143
Clade IIIa	<0.00000	-0.092
Clade IIIb	<0.00000	0.598
Clade IIIc	<0.00000	0.468
Clade IVa	<0.00000	0.146
Clade IVb	<0.00000	0.320
Clade IVc	0.00217	-0.030
Clade IVd	0.00019	0.072

Supplementary Figures

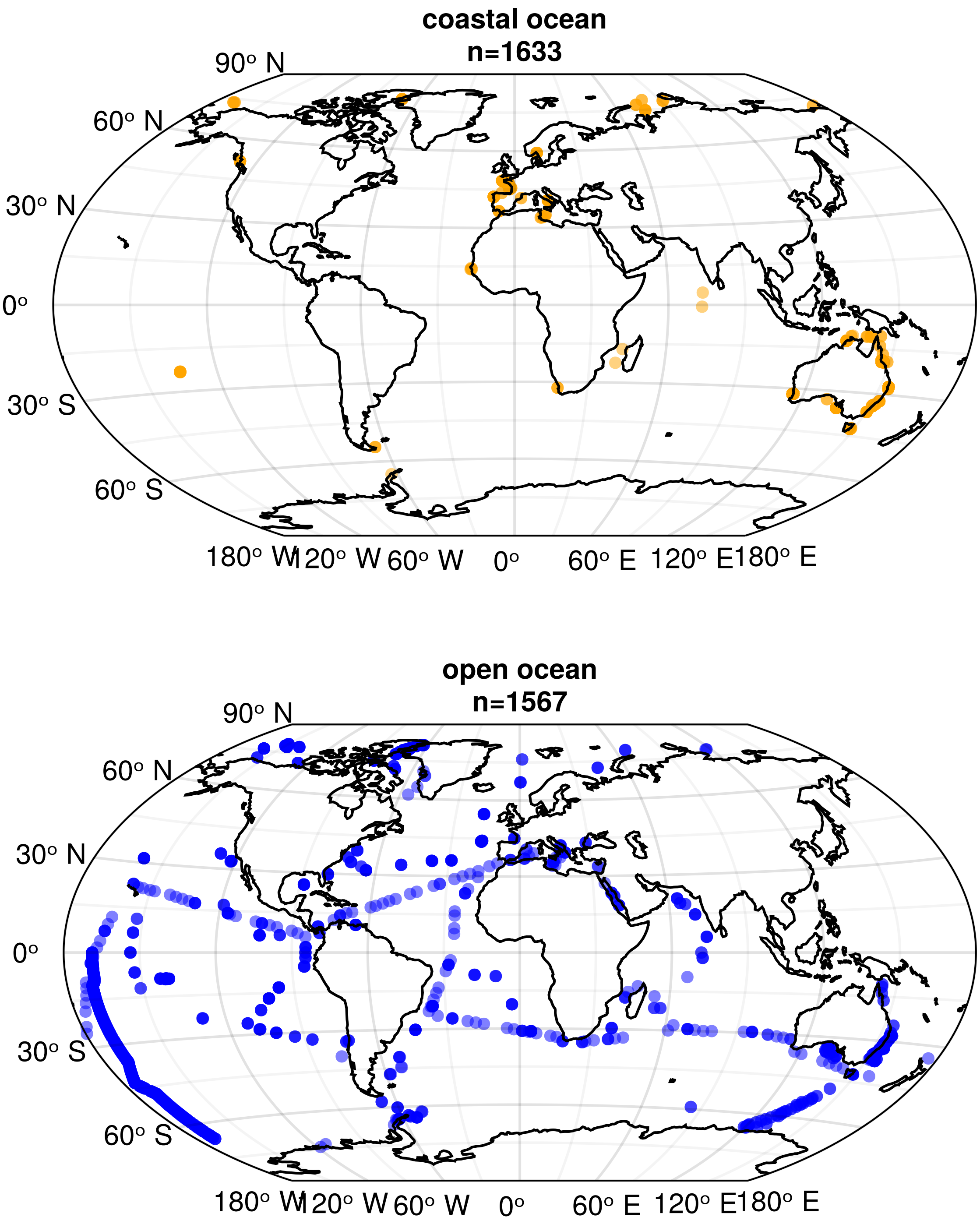

Fig. S1 | Geographic distribution of ocean samples selected from EukBank.

Orange dots represent coastal ocean samples and blue dots represent open ocean samples.

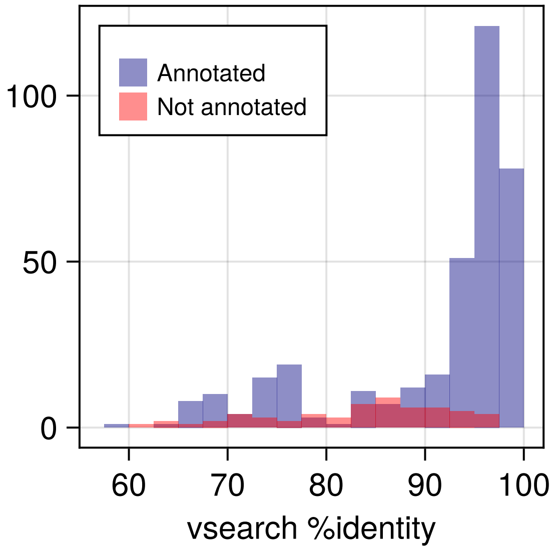

Fig. S2 | Distribution of identity against the best hit.

Blue bars represent ASVs annotated to the four clades, while red bars represent unannotated ASVs.

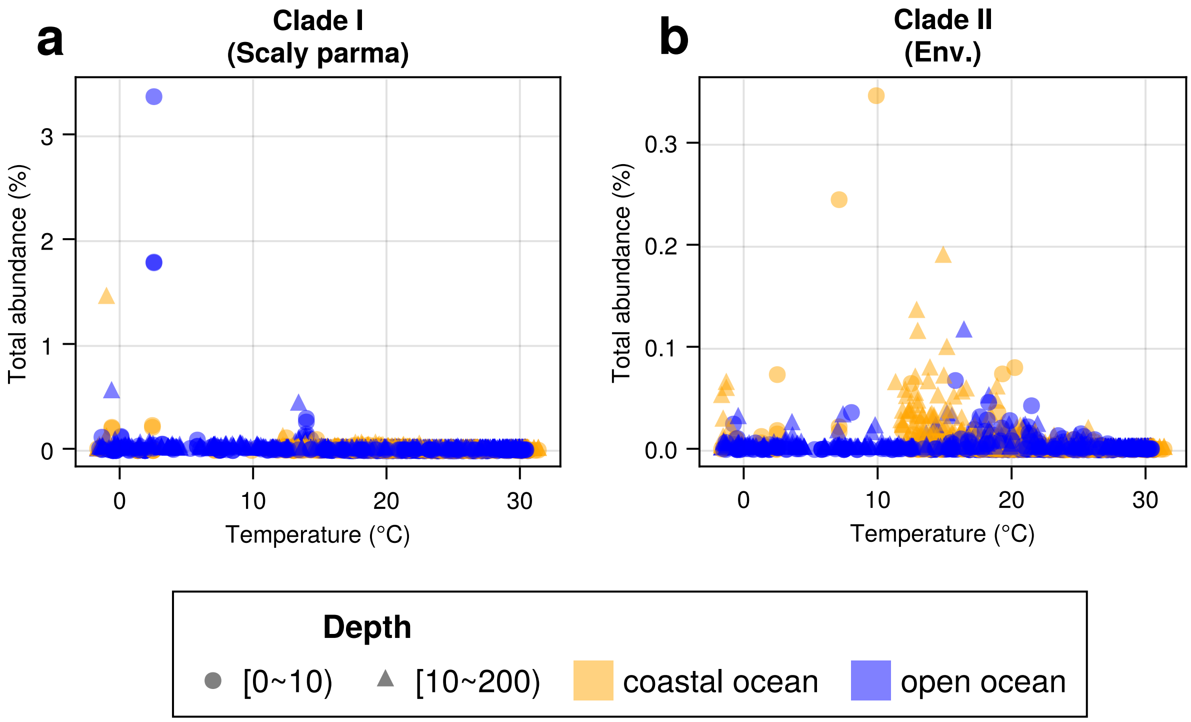

Fig. S3 | Distribution of total abundance across water temperature for Clade I and Clade II. Circles represent the surface (0-10 m), and triangles represent the euphotic zone (10-200m). Orange markers indicate coastal ocean samples and blue markers indicate open ocean samples. (a) Clade I ('Scaly Parma'), (b) Clade II (environmental clade).

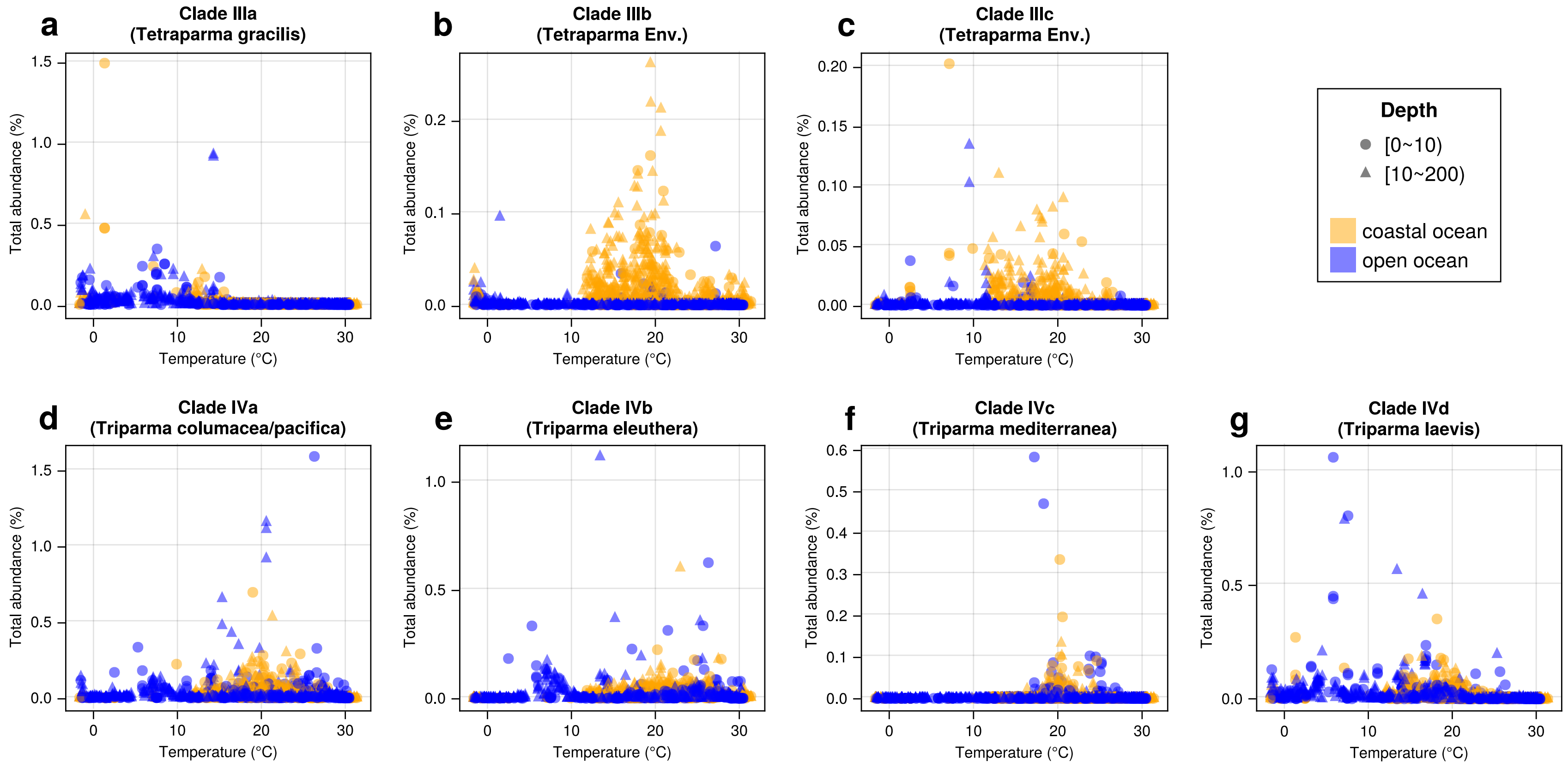

Fig. S4 | Distribution of total abundance across water temperature for each subclade of Clade III and Clade IV. Legend as Fig S3. (a) Clade IIIa (*Tetraparma gracilis*), (b) Clade IIIb (environmental clade), (c) Clade IIIc (environmental clade), (d) Clade IVa (*Triparma columacea*, *Triparma pacifica*), (e) Clade IVb (*Triparma eleuthera*), (f) Clade IVc (*Triparma mediterranea*), (g) Clade IVd (*Triparma laevis*).

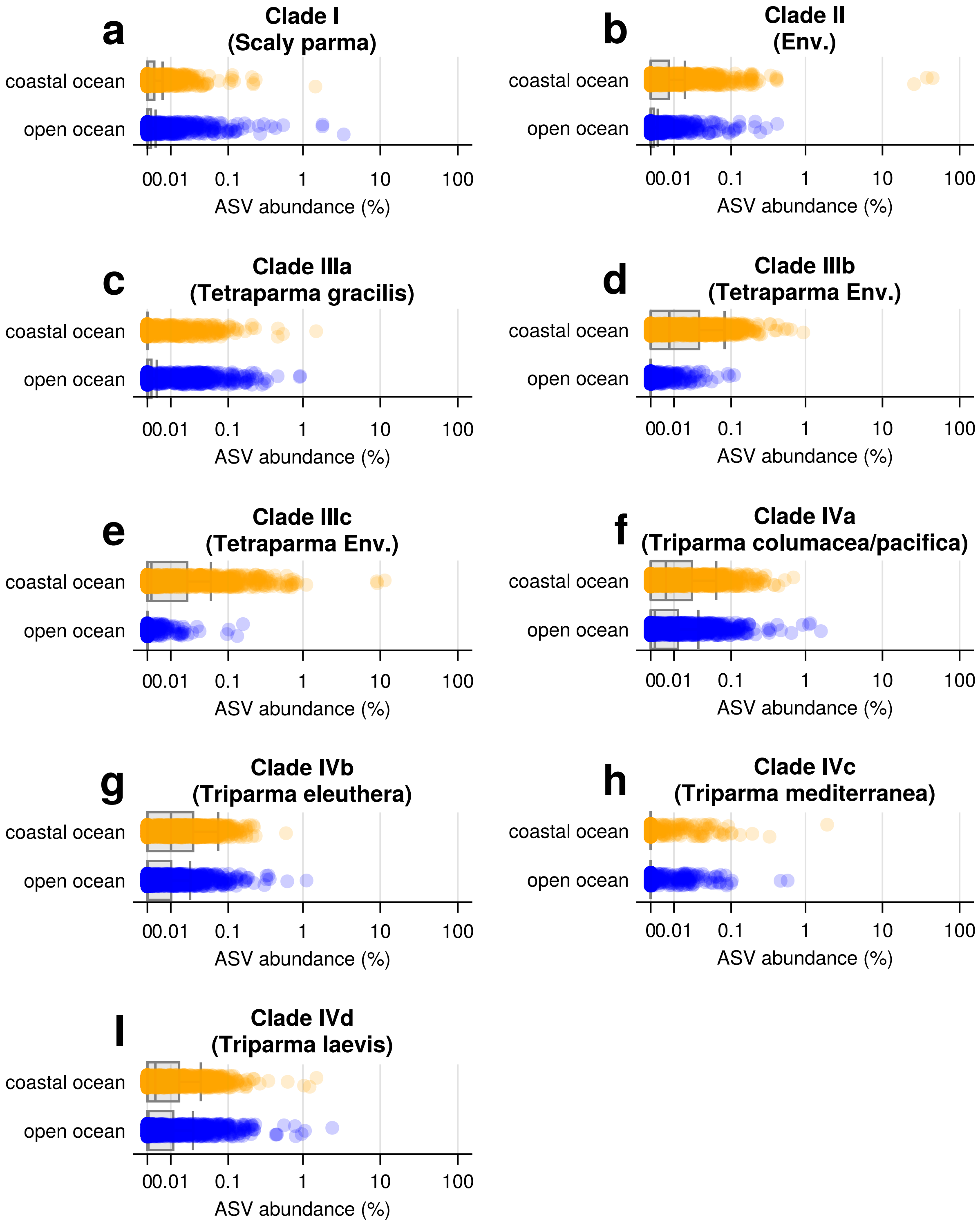

Fig. S5 | The distribution of total abundance in the coastal ocean and open ocean of each clade/subclade.

(a) Clade I (‘Scaly Parma’), (b) Clade II (environmental clade), (c) Clade IIIa (*Tetraparma gracilis*), (d) Clade IIIb (environmental clade), (e) Clade IIIc (environmental clade), (f) Clade IVa (*Triparma columacea*, *Triparma pacifica*) (g) Clade IVb (*Triparma eleuthera*), (h) Clade IVc (*Triparma mediterranea*), (I) Clade IVd (*Triparma laevis*).
